# Quantifying the scale effect in geospatial big data using semi-variograms

**DOI:** 10.1371/journal.pone.0225139

**Published:** 2019-11-14

**Authors:** Lei Chen, Yong Gao, Di Zhu, Yihong Yuan, Yu Liu

**Affiliations:** 1 Institute of Remote Sensing and Geographic Information System, School of Earth and Space Sciences, Peking University, Beijing, China; 2 Department of Geography, Texas State University, San Marcos, Texas, United States of America; The University of the South Pacific, FIJI

## Abstract

The scale effect is an important research topic in the field of geography. When aggregating individual-level data into areal units, encountering the scale problem is inevitable. This problem is more substantial when mining collective patterns from big geo-data due to the characteristics of extensive spatial data. Although multi-scale models were constructed to mitigate this issue, most studies still arbitrarily choose a single scale to extract spatial patterns. In this research, we introduce the nugget-sill ratio (NSR) derived from semi-variograms as an indicator to extract the optimal scale. We conducted two simulated experiments to demonstrate the feasibility of this method. Our results showed that the optimal scale is negatively correlated with spatial point density, but positively correlated with the degree of dispersion in a point pattern. We also applied the proposed method to a case study using Weibo check-in data from Beijing, Shanghai, Chengdu, and Wuhan. Our study provides a new perspective to measure the spatial heterogeneity of big geo-data and selects an optimal spatial scale for big data analytics.

## Introduction

Scale is a fundamental concept in geography [1]. It has a great impact on the representation, analysis, and aggregation of spatial data. Previous research [2–4] shows that nearly all geographical phenomena are scale-sensitive, which further highlights the significance of scale to geographic research. Openshaw [5] described this ‘scale-sensitive’ phenomenon as the modifiable areal unit problem (MAUP), which has two forms—the scale effect and the zoning problem. The scale effect refers to the fact that using coarser/finer analysis units will inevitably lead to different analysis results, whereas the zoning problem refers to the differences caused by the division of the study area even at the same spatial scale (e.g., dividing the study area into rectangles versus hexagons). This study focuses on investigating the scale effect. Although ‘scale’ is an ambiguous term with different semantic meanings, it is often used to refer to the size of the analysis unit or the spatial extent of the study area [6]. In spatial analysis, the size of units directly determines the amount of details to be included in the analysis and the results generated. This process creates the scale effect.

With the rapid development of information and communications technologies (ICTs), researchers in spatial science have access to a large amount of spatial data with high spatio-temporal resolutions. The significance of big geo-data on studying human mobility patterns and the socioeconomic environment has been widely recognized [7–9]. When conducting these urban-oriented studies, aggregating individual-level data into areal units is unavoidable, which raises the issue of choosing an appropriate scale when analyzing big geo-data. For example, in nationwide studies, it is common to use cities as the basic research unit. However, when it comes to urban-scale studies, there is no universally adopted analysis unit. In addition, many types of big geo-data used for urban studies, such as social media check-ins, are extensive data, where the size of the analysis unit determines the amount of data to be included and affects the value of each unit, whereas for intensive data, such as temperature and elevation data, the values are independent of the size of the analysis unit. For intensive data, the mean does not change with different analysis units and the variance declines when the analysis unit gets coarser [10], whereas for extensive data, the mean value changes when applying different analysis units and the variance can either increase or decrease with a coarser analysis unit. Therefore, analyses relying on extensive data are more scale-sensitive and it is more important to look into the scale effect of extensive data.

Previous work [11, 12] pointed out that the analytical results based on a single spatial scale cannot provide a complete view of the actual spatial patterns. One potential solution is to build multi-scale models [13–15] by aggregating the data at various scales. However, this solution only applies to simple data handling (e.g., spatial statistics), data storage, visualization, and sharing [15] due to its computational complexity. When discovering spatial patterns, researchers tend to arbitrarily choose one spatial scale for simplicity [8, 16–18]. For example, regular grids are mostly used for urban-oriented studies, where the sizes can vary from 200 m [16], 250 m [8], 500 m [17] to 1,000 m [18]. There is insufficient research on how to optimize the spatial scale in urban studies. Therefore, it is important to develop methods that can optimize the choice of spatial scale when characterizing and comparing aggregated data.

Similar research was also conducted by remote sensing scientists to select appropriate spatial resolutions for image processing [19]. The basic idea is that an appropriate spatial resolution can be determined by the spatial variation of land surface properties [19–24]. Various statistical measures, such as the local variance [20, 24], the variograms [21, 22], the semivariance at the lag of one pixel [23], and the scale variance [19], have been applied to solve this issue. Compared with these methods, a semi-variogram, as a commonly used analysis tool in geostatistics, provides measurable information regarding the variances of spatial units at different lags. Previous work has demonstrated the feasibility of semi-variograms to quantify spatial heterogeneity [25, 26] and explore spatial patterns at different scales [27]. For example, Garrigues et al. [25] constructed a normalize difference vegetation index (NDVI) variogram to quantify the spatial heterogeneity of land cover patterns. The univariate variogram model was extended by Garrigues et al. [26] to a multivariate variogram model that captures the spatial heterogeneity in both red and near infrared bands. Laush et al. [27] studied the effects of spatial and spectral scales on vegetation indices for different types of land cover.

Although the concepts are similar, there are fundamental differences between using semi-variograms to identify a suitable spatial resolution (i.e., spatial unit) for remote sensing imagery and using them for individual-level big geo-data. In remote sensing studies, researchers mainly focus on calculating the semi-variances between image cells to classify land use patterns and identify objects [19, 23]. Each image cell is considered a homogeneous unit, so it is not common to look into the variances within a certain image cell. However, individual-level big geo-data are often crowd-sourced point data, such as check-in data harvested from social media sites. Aggregating such point data to areal units inevitably leads to a loss of information. A simple example of aggregating point data is to divide a study area into grid cells, count the number of points in each cell, and use that count as the value of the cell. In this case, it is necessary to look into the variances of points within each cell (i.e., the intra-unit variance) to understand how much information was lost during aggregation. In addition, the cell values in remote sensing imagery are mostly intensive data (e.g., NDVI), meaning that the cell values are normalized and cannot be added up, whereas point-based big geo-data are extensive so the aggregated cell values can be added up [28]. The mathematical operations are different for these two types of data, therefore, the statistical measures for intensive remote sensing data cannot be directly applied to extensive spatial data. Based on the above reasons, our study proposes an innovative strategy to identify the optimal scale for extensive spatial data.

## Methods

### The semi-variogram and its key parameters

**Fig 1:**
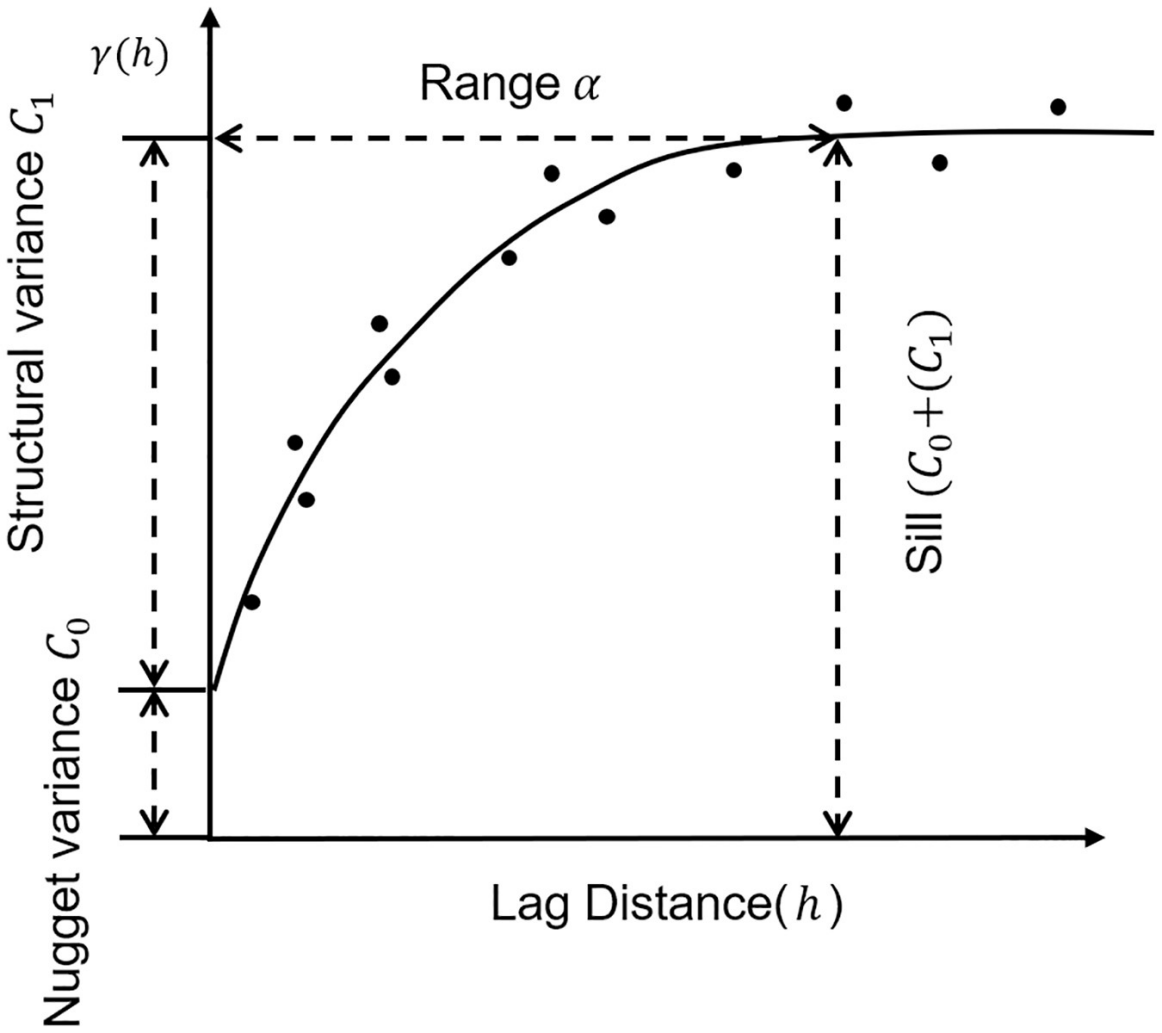
A diagram of the semi-variogram with three key parameters: The nugget variance (*c*_0_), the sill (*c*_0_ + *c*_1_), and the range of spatial autocorrelation (*α*).

As an efficient tool in geostatistics, a semi-variogram *γ*(*h*) was defined as half of the average squared difference of values between points separated at distance *h* [29], which is calculated as half a variogram. A set of *γ*(*h*) values can be obtained for each pairwise distance *h* as shown in Fig 1. The solid line represents a fitted semi-variogram (theoretical variogram) based on scattered semi-variance values (empirical variogram).

A semi-variogram is normally an increasing curve of the distance *h* (Fig 1) since nearby locations are more likely to be more similar than locations far apart. It consists of three main parameters [30], the nugget (*c*_0_), the sill (*c*_0_ + *c*_1_), and the range (*α*), reflecting different characteristics of spatial data variance.

As *h* increases, the semi-variogram may reach a steady point (i.e, the sill) or increase indefinitely. In previous studies, most variograms can reach the sill within the study area, otherwise the spatial variability of the data goes beyond the predefined spatial extent. The sill (*c*_0_ + *c*_1_) represents the total variation of the spatial dataset being investigated. As the partial sill, the structural variance (*c*_1_) reflects the intrinsic characteristic of data. The range (*α*), which is the distance where the variogram reaches the sill, representing the maximum spatial distance at which the dataset can still demonstrate spatial autocorrelation.

Theoretically, a variogram should go through the origin (0,0) because when the lag distance is getting close to 0, the differences between locations separated by this distance also approach 0. However, in practice, it is common for a variogram to not go through the origin and result in a positive intersect value at the y axis. This is called the ‘nugget effect’ and the non-zero intercept is the nugget variance (*c*_0_). It is can be caused by measurement errors or micro-variations that occur at a distance *h* smaller than the spatial granularity of the analysis [25]. In other words, the nugget variance (*c*_0_) either shows that there are errors during data collection, or locations separated at short distances still have substantially different values.

### Defining the nugget-sill ratio (NSR) for quantifying spatial data variance structures

There are three categories of spatial variations of aggregated data based on the relation between the lag distance and the size of the analysis unit: *intra*-*unit* *variation* if the lag distance is smaller than the size of the unit; *adjacent*-*unit* *variation* if the two are equal, and *inter*-*unit* *variation* if the lag distance is larger than the unit size. Intra-unit variation measures the information loss when aggregating point data into areal units, but it cannot be calculated directly. However, it is possible to use the *γ*(*h*) when *h* is smaller than the unit size to approximate the magnitude of the intra-unit variation. To this end, the nugget variance (*c*_0_) is a good indicator for estimating the intra-unit variation, because it is the lower limit of the intra-unit variation when *h* approaches 0. In addition, the nugget variance (*c*_0_) is scale-related, so it can be a useful indicator to quantify the intra-unit variation of aggregated spatial data at different scales. A large nugget variance indicates more substantial information loss during the aggregation. Therefore, researchers often prefer a small nugget variance if possible.

Geospatial big data, such as social media check-in data and taxi origin-destination data, are extensive and additive [28], meaning that the most common way to aggregate the data is using a simple sum operation. This aggregation process inevitably introduces an increasing intra-unit variation and a larger nugget variance (*c*_0_) at a coarser scale. One solution to compare indicators at different scales is to make extensive data intensive [28] (e.g., convert population to population density). Another solution is to use a normalized and scale-free indicator. In this work, we adopt the nugget-sill ratio *c*_0_/(*c*_0_ + *c*_1_) (NSR) as the measure of spatial data variance structures. The NSR contains information about both the intra-unit variation (i.e., the nugget variance) and the inter-unit variation, as the sill (*c*_0_ + *c*_1_) is an approximation of the limit of the inter-variation; therefore, it is an appropriate indicator of the scale effect in this study.

The NSR refers to the ratio of the micro-variance as opposed to the total variance [31]. When point data are aggregated to areal units, attributes of all points within the same unit are represented by the aggregated attributes of the unit. This inevitably causes information loss. Previous studies [31–33] used this indicator to characterize the spatial dependency between locations, i.e., a smaller NSR shows a stronger spatial dependency. It is based on the principle that the closer two locations are, the more similar attributes they have. This study uses the NSR to quantify the information loss and the scale effect when aggregating point data.

### Estimating semi-variances and calculating the NSR

Eq (1) is used to estimate the semi-variance $\hat{\gamma }\left(h\right)$. We use regular cells as the spatial unit, and the cell size represents the scale of aggregation. In Eq (1), *x*_*i*_ and *x*_*i*_ + *h* are cells separated at distance *h*; *z*(*x*_*i*_) is the value of cell *x*_*i*_, which is the sum of properties of all points within that cell; *N*(*h*) is the number of pairs of cells located distance *h* from each other.

$$\hat{\gamma }\left(h\right)=\frac{1}{2N(h)}\sum_{i=1}^{N(h)}{\left[z\left(x_{i}\right)-z\left(x_{i}+h\right)\right]}^{2}$$ (1)

Considering that the semi-variance value is not statistically reliable at large distances due to the decreasing number of cell pairs *N*(*h*) [30], we chose to only calculate the semi-variance for lag distances smaller than half of the extent of the study area [30, 34]. We also equally divided the x-axis into several ranges of lag distances (instead of using a specific distance) to make sure that we had enough grid pairs in each range. Therefore, Eq (1) is converted to the following format:

$$\hat{\gamma }\left(d_{k}\right)=\frac{1}{2N(k)}\sum_{l=1}^{N(k)}{\left[z\left(x_{1}^{l}\right)-z\left(x_{2}^{l}\right)\right]}^{2}$$ (2)

where *k* is the index of a given distance range (i.e., a ‘distance bin’) and *d*_*k*_ is calculated as a representative distance of the *k*th distance range as defined in Eq (3); *N*(*k*) is the number of cell-pairs within the *k*th distance range; $|x_{1}^{l}-x_{2}^{l}|\in \left[h_{k}-\epsilon ,h_{k}+\epsilon \right]$, where *h*_*k*_ is the median of *k*th distance bin, *ϵ* is a distance tolerance, $x_{1}^{l}$ and $x_{2}^{l}$ represent the *l*th cell pair within the distance bin and $z(x_{1}^{l})$, $z(x_{2}^{l})$ are values of cells $x_{1}^{l}$ and $x_{2}^{l}$, respectively.

$$d_{k}=\frac{1}{N(k)}\sum_{l=1}^{N(k)}\left|x_{1}^{l}-x_{2}^{l}\right|$$ (3)

where $|x_{1}^{l}-x_{2}^{l}|$ represents the Euclidean distance between the cell-pair ($x_{1}^{l},x_{2}^{l}$) and *d*_*k*_ is the mean value of pairwise distances between all cell pairs in the *k*th distance bin.

According to Eq (2), we can obtain a series of discrete semi-variance estimates, but to calculate the NSR, we still need to fit a continuous mathematical model to the empirical semi-variogram. These models are usually selected from a set of predefined functions [3, 35], which ensures that the predicted variances are non-negative, such as the Gauss, spherical, exponential, and power models. Although the polynomial model is not included in these models, it is selected with the Gauss model in this study, as they can match the shape of empirical variograms and also follow the aforementioned ‘non-negative’ principle. The Gauss is defined:

$$\gamma \left(h\right)=c_{0}+c_{1}\left(1-e^{-\frac{h^{2}}{d^{2}}}\right)$$ (4)

where *d* is the distance parameter; the practical range is $\sqrt{3}d$ [34]. The polynomial model is

$$\gamma \left(h\right)=-\frac{c_{1}}{a^{2}}h^{2}+\frac{2c_{1}}{a}h+c_{0}$$ (5)

where *a* is the range (*α*).

### Research process

**Fig 2:**
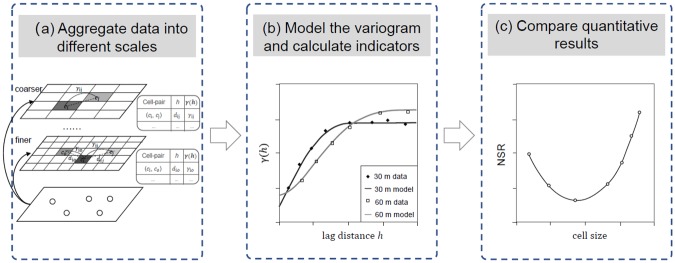
Process of quantifying the scale effect with a semi-variogram.

Fig 2 shows a scale-adaptive integrated method for scale effect evaluation with three steps: (a) aggregating point data into areal units under multiple scales; (b) fitting the empirical variogram models and calculating indicators; and (c) comparing quantitative results.

First, we aggregate discrete point data into cell units with different sizes. Values of discrete points within each cell are accumulated as the attribute value of that cell. For example, the number of check-ins in each cell is the sum of check-ins from all discrete points in that cell. We then estimate semi-variances for each cell pairs in the study area.

Second, we fit the empirical variogram and calculate the NSR. For aggregated data at each scale, we take the same steps as follows: Before estimating semi-variance values, we need to decide the distance tolerance *ϵ*. To investigate the scale effect, we assume that the *ϵ* is only proportional to the cell size, and define that the number of cell pairs within each distance bin should be more than 30 as suggested by Huijbregts [34]. After calculating the discrete semi-variogram estimates, we fit the Gauss and polynomial models and then calculate the NSR.

Finally, we plot a diagram showing the correlation between the NSR and the scale. A small NSR value means that the intra-cell variance accounts for a small percentage of the total variance, and thus it can be considered as a guide to the optimal scale (*S*_*o*_). More detailed analyses are presented in the ‘experiments with synthetic data’ section and the ‘case study’ section.

In general, this method builds a bridge between scales and observed spatial data and then quantifies the scale effect by comparing a group of indicator values at different scales.

## Experiments with synthetic data

**Fig 3:**
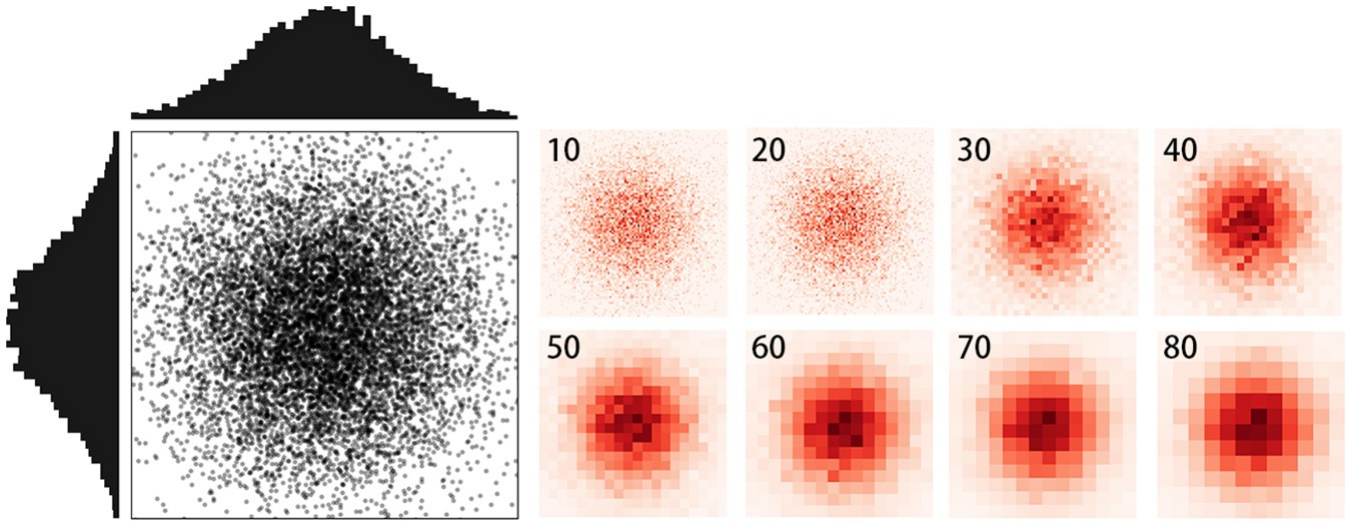
Generated point data follow a two-dimensional normal distribution. The right panel shows the corresponding heat maps.

We designed two sets of simulation experiments to verify the feasibility of the method. The simulated data in this section are scale-free. The entire study area is a square-shaped 1,000 × 1,000 areal unit. The cell sizes were selected from 10 to 80 with a 5-unit interval. We generated simulated points from a two-dimensional normal distribution N(*μ*_1_, *μ*_2_, *σ*_1_, *σ*_2_, *ρ* = 0). Centers of the generated points were fixed to the center of the study area (*μ*_1_ = *μ*_2_ = 500), and *σ* (*σ*_1_ = *σ*_2_ = *σ*) is a variance parameter representing the spatial dispersion of the simulated data. In addition, *N* denotes the number of discrete points generated. Fig 3 shows the spatial distribution of the generated points and the corresponding heat maps at various scales. At a finer scale, each unit is small and more similar to each other. As the scale gets coarser, it is easier to see the spatial heterogeneity of different cells. We conducted two simulations with different *N* and *σ* to test the proposed method.

**Fig 4:**
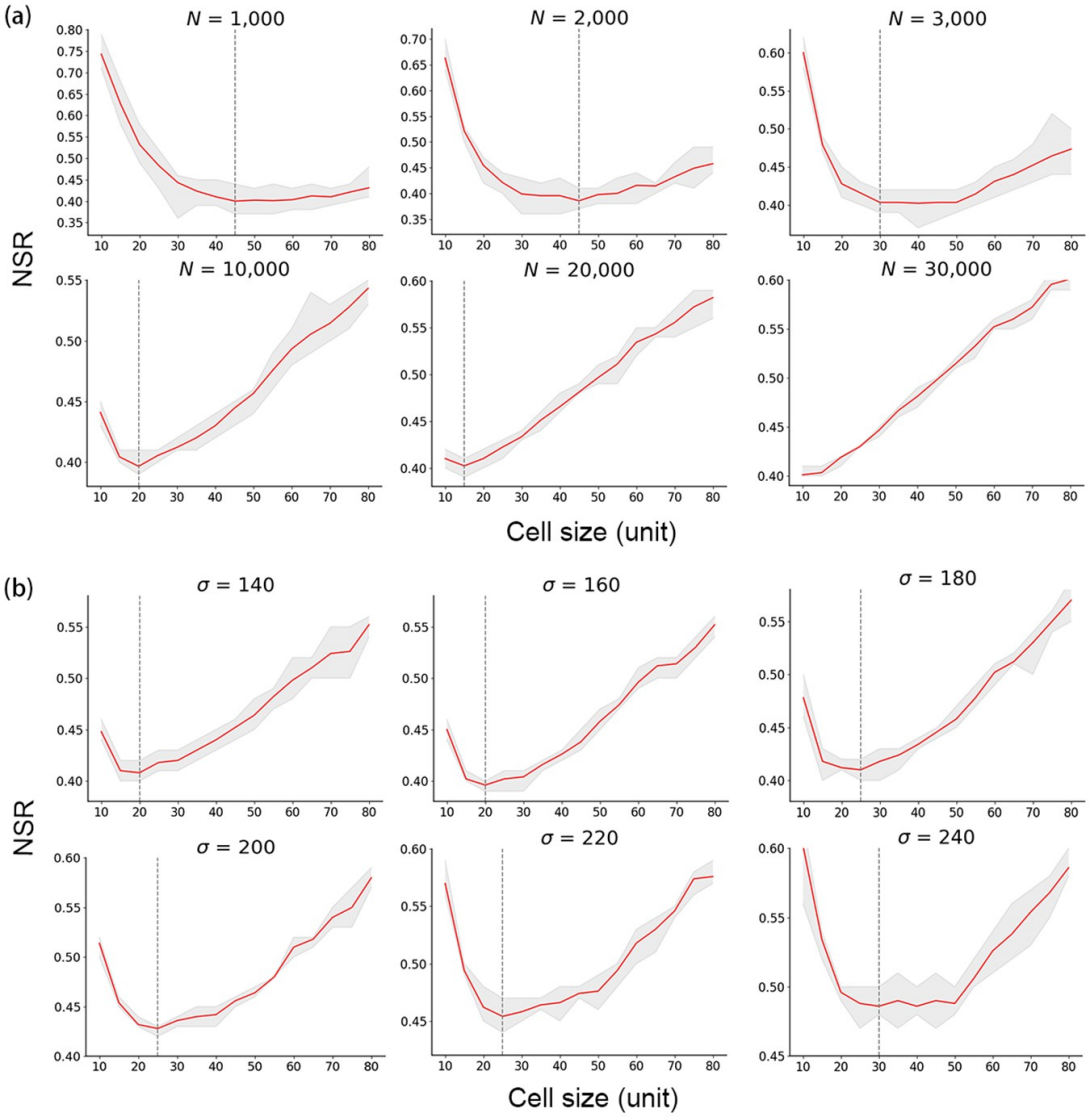
Correlation between the NSR and the cell size. (a) *σ* = 150 and *N* varies; (b) *N* = 10,000 and *σ* varies. The gray area represents the range of multiple experimental results and red lines mark the average value. The dotted lines mark the first local minimum NSR.

Setting *σ* = 150 ensures that 99% of the generated points can be included in the study area. We set the number of points *N* as 1,000, 2,000, 3,000, 10,000, 20,000, and 30,000 to test different parameter settings. To ensure reliable results, we generated data with the same parameters 20 times and calculated their average NSR. The correlation between the NSR and the cell size are shown in Fig 4(a). Except for the last sub-figure where *N* = 30,000, the other sub-figures all show a U-shaped curve, where the NSR first decreases and then increases when the cell size increases (i.e., when scale gets coarser).

At the finest scale (cell szie = 10), the whole study space is divided into fragmented small units. Each cell only contains a small number of points. As a result, the intra-cell variation and the inter-cell variation are very close, resulting in the NSR approaching 1. It is similar to the ‘pure nugget effect’ discussed in [3], where the semi-variance shows similar values at all lags. The differences between cells are gradually revealed when the cell scale increases. When the scale gets too coarse, the spatial information inside each cell is highly generalized, and the variance of cell values decreases. The intra-cell variation is again getting close to the inter-cell variation, which leads to an increase in the NSR. That proves that the NSR is effective in characterizing the structure of spatial variances.

We defined the optimal scale (*S*_*o*_) as the cell size when the NSR reaches the first local minimum. Based on this definition, *S*_*o*_ = 45, 45, 30, 20, and 15 where *N* = 1,000, 2,000, 3,000, 10,000, and 20,000, respectively. However, we cannot find an *S*_*o*_ corresponding to when *N* = 30,000, since the scale is less than the minimum scale (10) we considered. The *S*_*o*_ values get smaller with an increasing *N*, which indicates the impact of data density on the scale effect.

Based on the results from the previous step, we set *N* = 10,000 and explored the role of *σ* in determining the scale effect. The six graphs in Fig 4(b) demonstrate a similar U-shaped curve, which is consistent with results from the previous step, and *S*_*o*_ = 20, 20, 25, 25, 25, and 30 when *σ* = 140, 160, 180, 200, 220, and 240, respectively. As *σ* increases, the minimum NSR appears at a coarser scale, which implies that *σ* also has an impact on the scale effect. In other words, when aggregating data with a larger variance, each analysis unit naturally contains more information compared to when the variance of the data is smaller, thus it is preferable to use a finer analysis unit. On the contrary, when *σ* is smaller, the corresponding analysis unit should be coarser.

## Case study

### Data description

**Fig 5:**
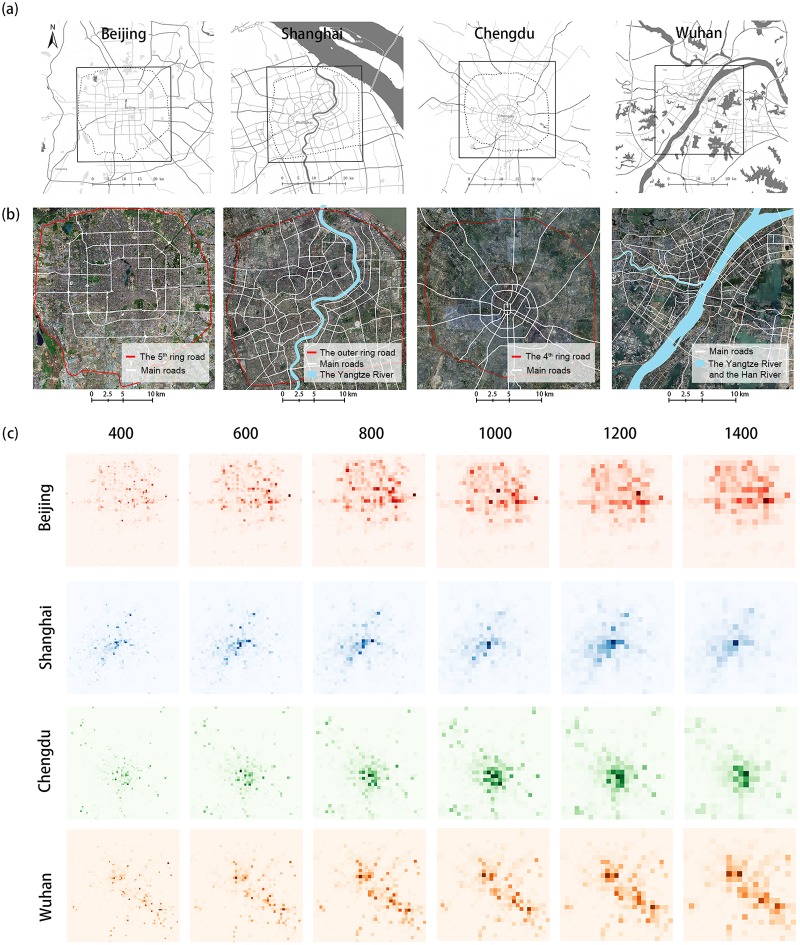
The study areas in Beijing, Shanghai, Chengdu, and Wuhan. (a) the study areas; (b) satellite imagery; (c) spatial distributions of check-in data at varying scales in these cities.

In addition to the simulated experiments, we selected two major cities, Beijing and Shanghai, and two medium-sized cities, Chengdu and Wuhan, to test the methodology. Because human activities are mainly concentrated in the urban area of these cities, we defined the study areas with the same dimensions (30 km by 30 km) in all cities (Fig 5(a)). The study area of Beijing covers the districts within the 5^*th*^ ring road and the study area of Shanghai covers the central area within the outer ring road. For Chengdu, the study area covers the area within the 4^*th*^ ring road and the study area of Wuhan consists of the area within 3^*th*^ ring road and partial regions within the 4^*th*^ ring road.

To mitigate the zoning effect, we used regular grids as the analysis unit in this case study, with cell sizes from 300 m to 2,000 m with a 100 m interval. The actual study area may be slightly smaller than 30 km × 30 km, as the number 30 km is not divisible by certain cell sizes (e.g., 700 m, 800 m, etc) (Table 1).

**Table 1:** Cell sizes and the corresponding basic attributes.

Cell size	Cell Number	Row	Column
300 m×300 m	10,000	100	100
400 m×400 m	5,625	75	75
500 m×500 m	3,600	60	60
⋯			⋯
1,800 m×1,800 m	256	16	16
1,900 m×1,900 m	225	15	15
2,000 m×2,000 m	225	15	15

We used the point of interest (POI) check-in data in Beijing, Shanghai, Chengdu, and Wuhan from Sina Weibo in 2014. Sina Weibo is the biggest microblog service in China functionally similar to Twitter. We collected our dataset using the official Weibo Application Programming Interface (API) (https://open.weibo.com/wiki/2/place/nearby/pois). For each POI, the record includes its place name, address, geographical coordinates, and the number of check-ins at this POI. After data filtering, we obtained 88,886, 78,864, 26,907, and 24,542 valid records for Beijing, Shanghai, Chengdu, and Wuhan, respectively.

We calculated the number of check-ins within each cell at different scales. Fig 5(c) shows the heat maps of check-ins at varying spatial scales. As can be seen, the spatial distribution of check-ins demonstrates very different patterns in each city. In Beijing, the data show a polycentric pattern where there are multiple clusters of check-ins in different parts of the city. Beijing’s polycentric urban activity pattern has been discussed in many previous studies [36, 37]. In addition, there are more check-ins in the north than in the south. This is potentially because the northern side of Beijing is more developed with better facilities and infrastructures [38]. Unlike Beijing, Shanghai shows a monocentric pattern [39, 40] where the check-ins are concentrated in the southwest of the study area (i.e., the central urban area of Shanghai). For Chengdu, cells with a higher check-in density are mainly clustered in the center and to the south of the city, and there are a few high density cells in the outer areas of the city. Check-ins in Wuhan show a morphologically polycentric pattern [41], which is potentially due to its complex configuration of water bodies. As shown in Fig 5(b), the Yangtze River and the Han River divide the urban center of Wuhan into three sections. There are also a large number of lakes and other water bodies that contribute to the discontinuity of the central urban area in Wuhan.

In addition, the spatial distribution of check-ins varies at different scales. At a finer scale, high-value cells scatter across the whole study area, whereas at a coarser scale, high-value cells are more clustered. For example, the heat map of Shanghai when the cell size equals 1,400 m shows a monocentric pattern; however, at other scales, the heatmaps show a polycentric pattern.

### Quantitative results of the scale effect

As mentioned in the methodology, we used the NSR to quantify the scale effect of POI check-ins in the four cities. We applied several strategies to ensure the robustness of the experiment design. First, to mitigate the impact of the actual study area, at each scale, we chose ten slightly different 30 km × 30 km study areas within each city and calculated the average NSR. Second, we tested different models that can be used to fit into the estimated semi-variance values, and we chose the polynomial model due to its better fitting performance. The fitted models were evaluated based on the goodness of fit *R*^2^, which is a commonly used indicator to measure the performance of a statistical model.

**Fig 6:**
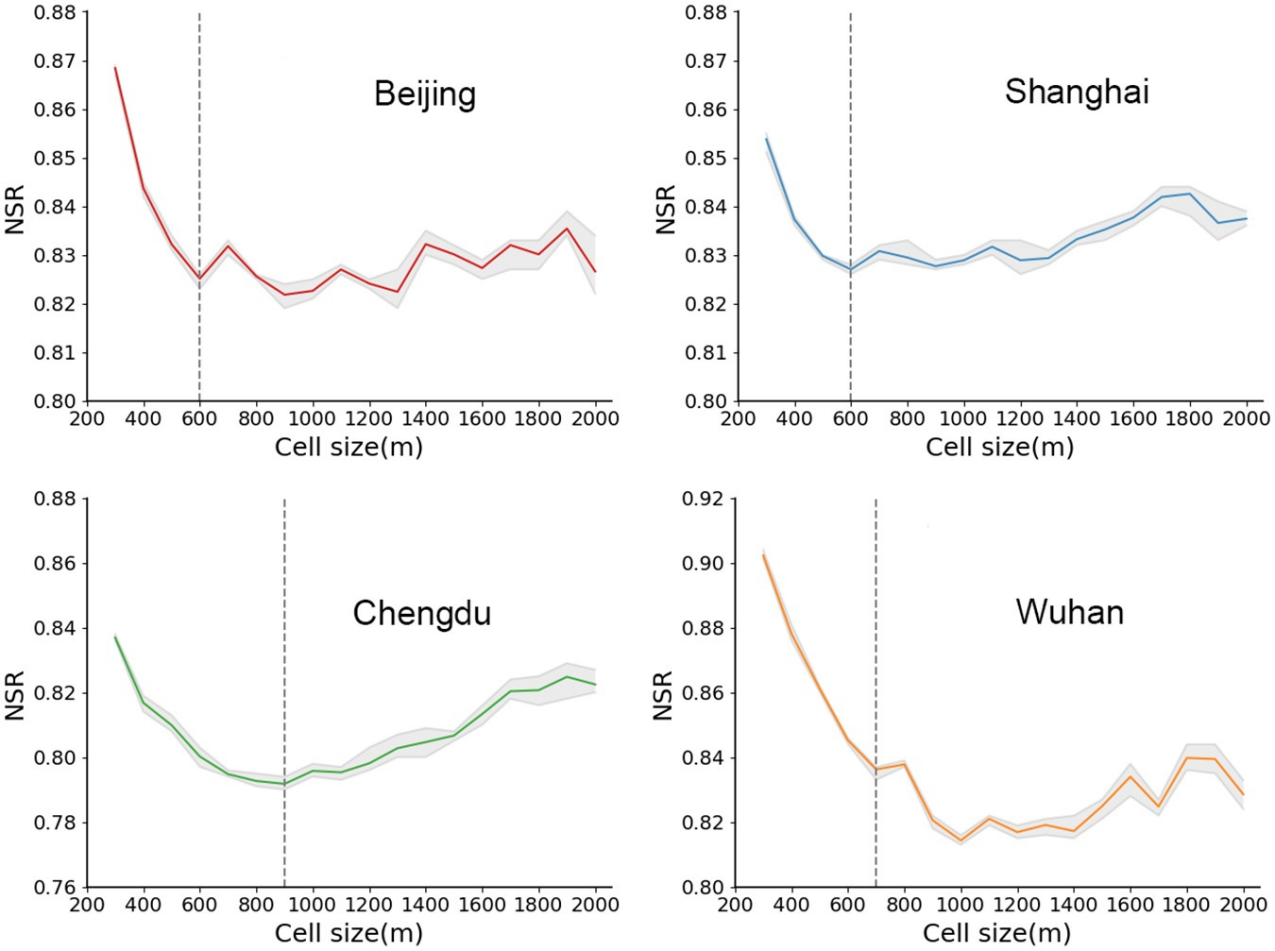
The correlation between the NSR and the cell size in Beijing, Shanghai, Chengdu, and Wuhan. The gray shades represent the range of multiple runs. The solid lines mark the average value of all experiments.

Fig 6 illustrates the correlation between the NSR and the spatial scale in the four Chinese cities. As can be seen, all four curves are U-shaped and the maximum NSR values appear at the finest scale (i.e. 300 m). The optimal scale *S*_*o*_ for Beijing, Shanghai, Chengdu, and Wuhan are 600 m, 600 m, 900 m, and 700 m, respectively. Due to the differences in city sizes and the amount of data, we will compare two large cities (Beijing and Shanghai) and two medium-sized cities (Wuhan and Chengdu) separately to better interpret Fig 6.

As shown in Fig 6, Beijing has a slightly larger NSR range than Shanghai, indicating that the spatial characteristics of Beijing are more affected by spatial scales.

Both curves can be divided into two parts with 600 m as the cut-off point. The former half of both curves show a rapid decline, but the latter half indicates an increasing trend. Specifically, the NSR in Shanghai first drops rapidly and then rises slowly with slight fluctuations; while the NSR has more fluctuations in Beijing after the first local minimum. This suggests that urban configuration of Beijing is more complex than that of Shanghai. For Chengdu, the NSR values are generally lower than those in Wuhan. This shows that POI check-in data in Chengdu are more spatially dependent. Moreover, it should be noted that the NSR for Wuhan shows a more fluctuated pattern. This is probably because check-in data in Wuhan demonstrate a more dispersed pattern due to the large number of lakes and ponds in Wuhan (Fig 5(b)). In addition, the *S*_*o*_ for Chengdu and Wuhan are coarser than for Beijing and Shanghai, which further demonstrates the impacts of data density on the scale effect discussed in the experiments using synthetic data.

### Evaluation of the optimal scale *S*_*o*_

To further evaluate the results, we introduced an indicator (the homogeneity within a cell, denoted by *Hom*) based on the q-statistic [42, 43]. Q-statistic is a statistical method for measuring the degree of spatial stratified heterogeneity and uncovering its possible determinants [42, 43]. Spatial stratified heterogeneity refers to the phenomenon that occurs when dividing a study area into sub-regions, the within region variance is smaller than the between region variance. A typical example of the spatial stratified heterogeneity is the differences between climate zones. The division of the sub-regions inevitably affects the degree of spatial stratified heterogeneity. In the scope of this study, a smaller *Hom* represents a greater spatial stratified heterogeneity, which further indicates a more homogeneous pattern within the sub-regions (units) and less information loss during the aggregation. It provides a feasible measure for validating whether the obtained optimal scale, *S*_*o*_, corresponds to the least amount of information loss.

The *Hom* indicator is defined as:

$$Hom=\frac{SVI}{SVT}=\frac{\sum_{i=1}^{n}N_{i}\sigma_{i}^{2}}{N\sigma^{2}}$$ (6)

where $\sigma_{i}^{2}$ is the variance of POI check-ins within cell *i* and *σ*^2^ is the variance of all POI check-ins; *N*_*i*_ is the number of POIs within cell *i*, *N* is the number of all POIs, i.e. $N=\sum_{i=1}^{n}N_{i}$. *SVI* and *SVT* are the sum of intra-unit variances and the total sum of variances, respectively. For given spatial point data, *SVT* is fixed while *SVI* varies with the scale.

The *Hom* indicator ranges from 0 to 1. It represents the degree of intra-cell variability at different scales, which is reflected by not only the number of points within an analysis unit, but also the variance of attributes at these points. A lower *Hom* indicates that the analysis units are more homogeneous internally and the intra-unit variances are lower, and therefore we have less information loss. As the scale gets coarser, the number of POIs within each cell increases. This results in an increase in intra-cell variability. Moreover, spatial point patterns can be a result of a complex urban configuration or multiple spatial processes, thus there may be more than one local minimum value of the *Hom* indicator (i.e., the optimal scale). In this paper, we adopt the same idea as the elbow method [44] when deciding the number of clusters and use local minimums to identify the optimal scale based on the *Hom* indicator (*S*_*hom*_).

**Fig 7:**
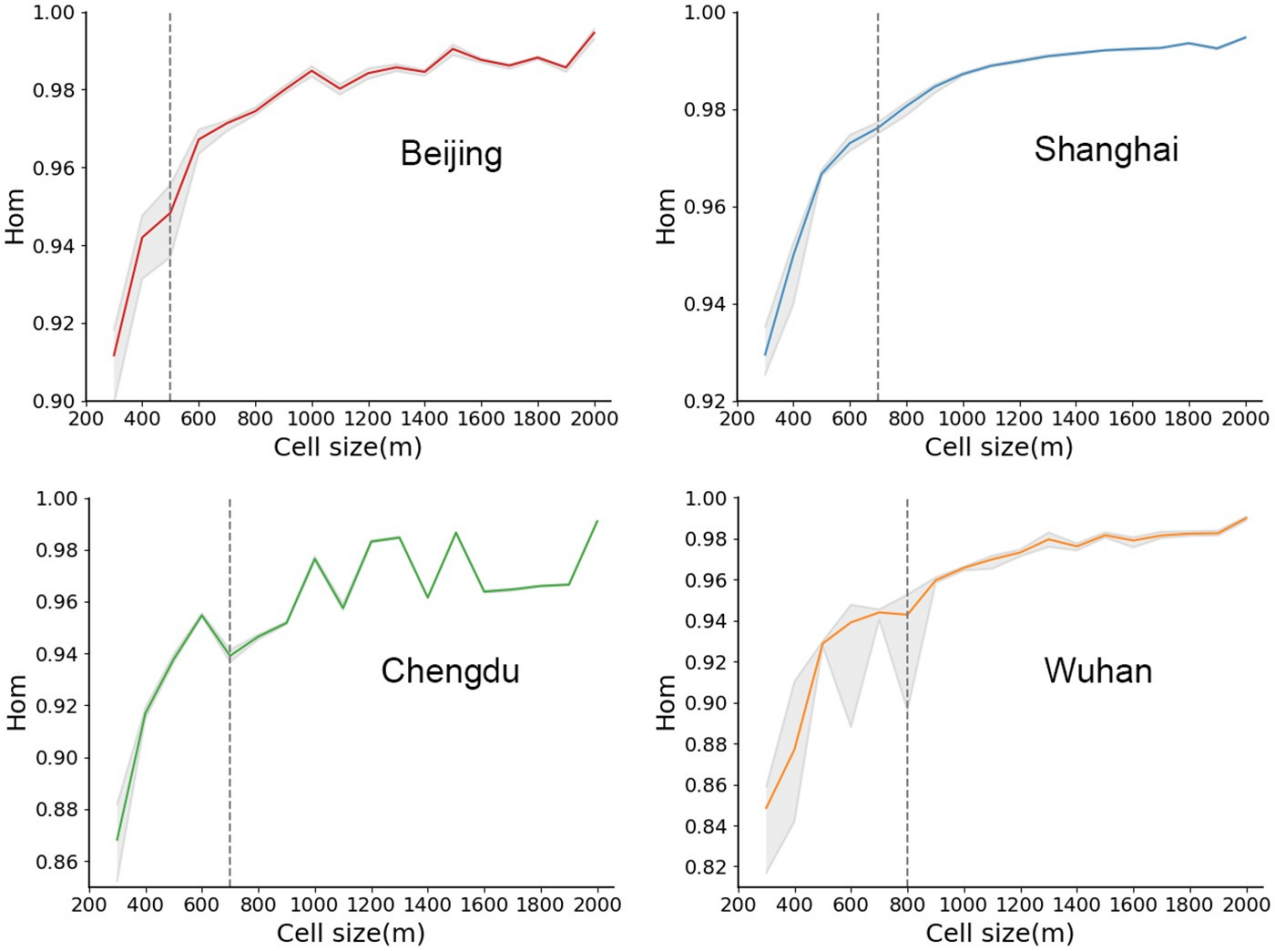
Evaluation of optimal scales based on the *Hom* indicator. The dotted lines mark the *S*_*hom*_ value, which is the elbow point when the scale is optimized.

Fig 7 shows the evaluation results for four Chinese cities. According to these results, we can conclude that for Beijing, Shanghai, Chengdu, and Wuhan, the *S*_*hom*_ values are 500 m, 700 m, 700 m, and 800 m, respectively. These results are slightly different from the *S*_*o*_ values calculated from the NSR because the *Hom* indicator only measures the intra-unit variability and does not consider the inter-unit variability. In other words, at each given scale, if we randomly switch the locations of cells, the *Hom* indicator remains constant, because the parameters in Eq (6) (i.e., *N*, *N*_*i*_, *σ*, $\sigma_{i}^{2}$) do not change as long as the cells are still divided the same way. The city with the biggest difference between the *S*_*hom*_ and the *S*_*o*_ values is Chengdu. For other cities, the optimal scales recommended by the NSR and the *Hom* indicator are very similar. In addition, the orders of the *S*_*o*_ and the *S*_*hom*_ values for these cities are consistent. Both the *S*_*o*_ and the *S*_*hom*_ values for Beijing and Shanghai are smaller than those for Chengdu and Wuhan. This further validates the robustness of our method.

## Discussion

### Influence of different fitting models

**Fig 8:**
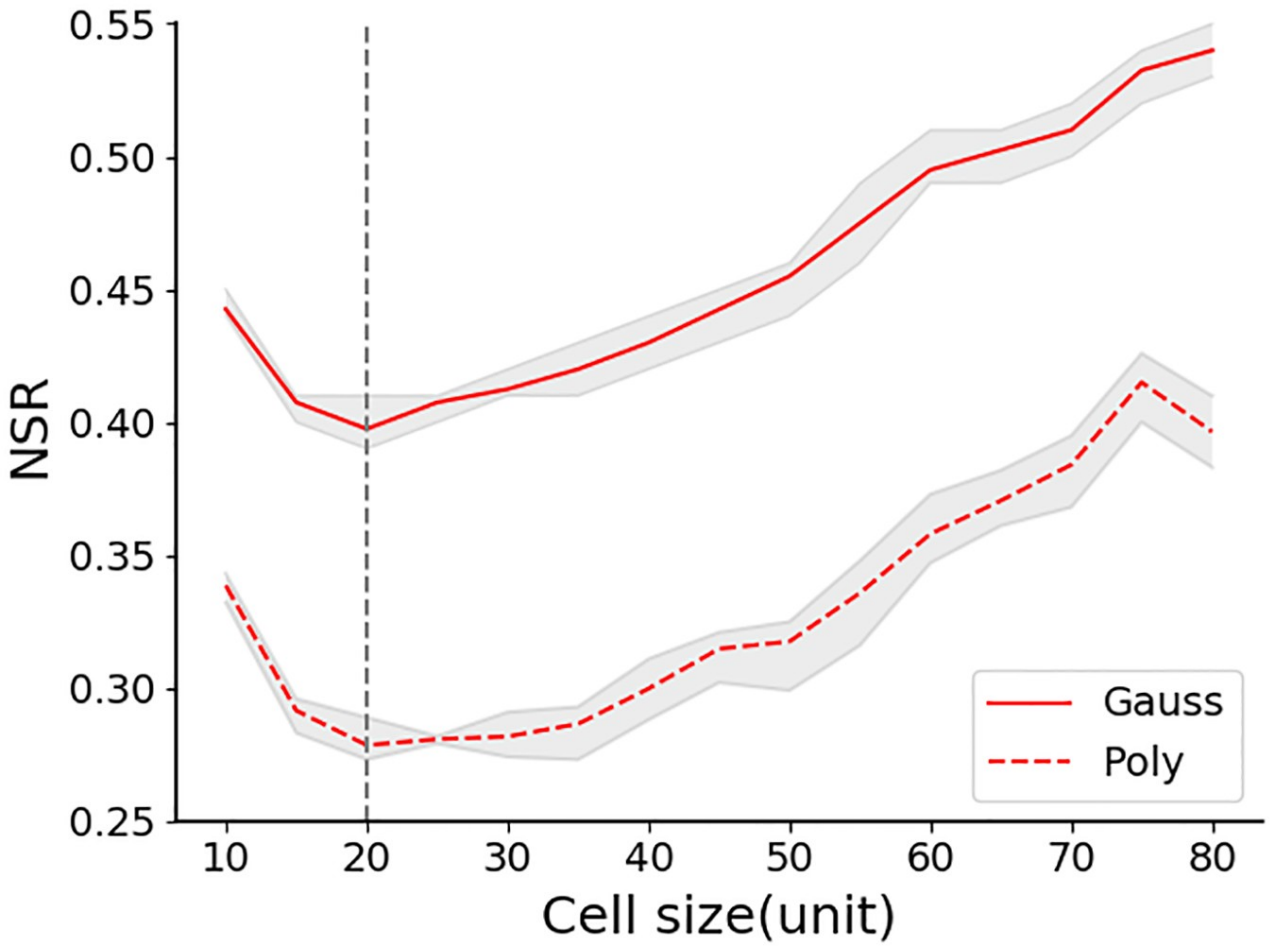
Influence of different fitting models on the NSR. Data used are single-centered data in the ‘experiments with synthetic data’ section where N = 10,000, *σ* = 150. The solid and dashed lines represent results fitted by the Gauss and polynomial models, respectively.

Considering that fitting different models to the same semi-variogram may lead to different results, we adopted multiple models to fit the same data to investigate how the model selection may impact the scale effect. We employed the polynomial model and the Gauss model to fit the simulated data in the ‘experiments with synthetic data’ section 3 to compare the results. Because the results are similar, we only discuss one set of data (*N* = 10,000, *σ* = 150) as an example. As illustrated in Fig 8, the solid and dashed lines are results fitted by the Gauss and polynomial models, respectively. It can be seen that the NSRs fitted by the Gauss model are generally higher than those fitted by the polynomial model, which is potentially due to the different characteristics of the models. Although the absolute values are different, both curves follow the same trend. In addition, the optimal scale *S*_*o*_ values are consistent. These results show that the choice of models does not have a substantial impact on the results in our analysis.

### Experiments with dual-centered data

**Fig 9:**
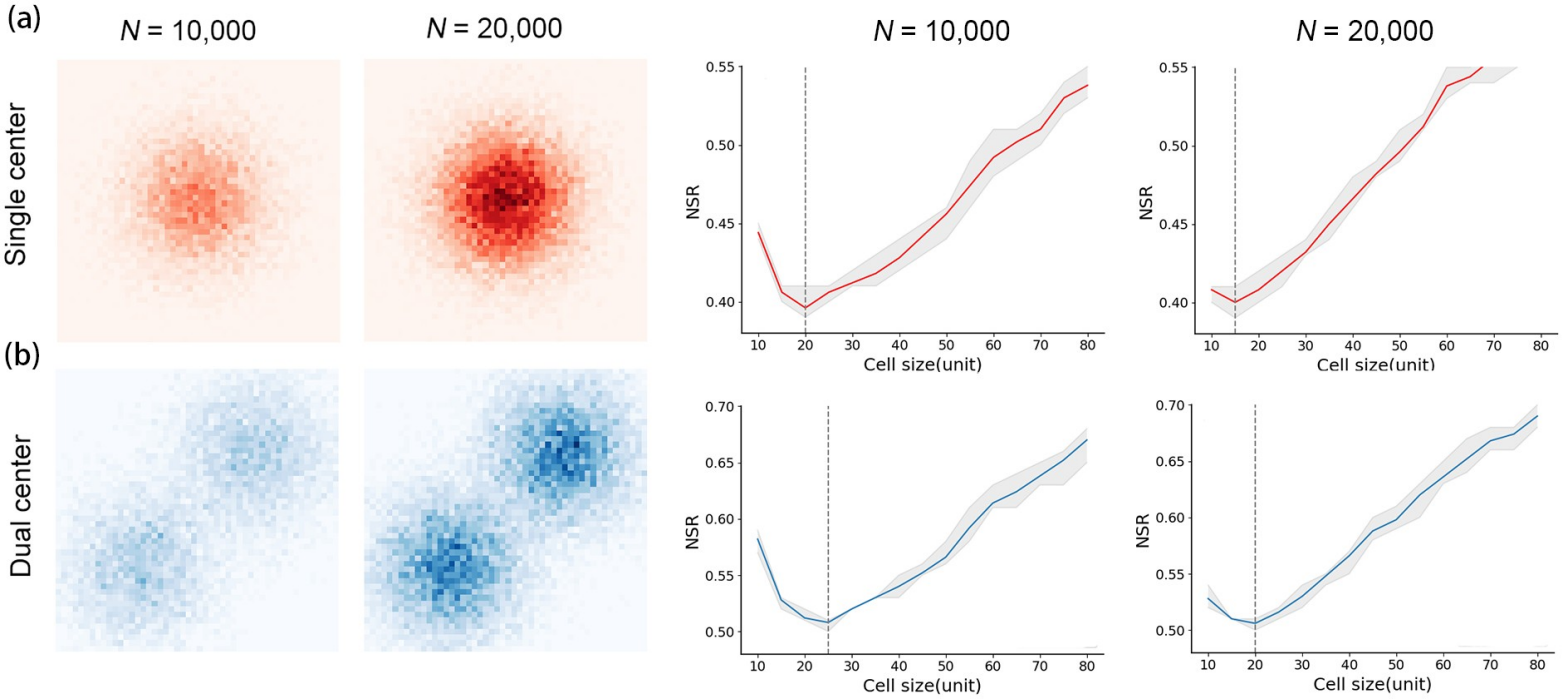
Spatial distribution of simulated data and the corresponding results of the scale effect. (a) single-centered data; (b) dual-centered data. *N* represents the number of points for each center.

The patterns in the ‘experiments with synthetic data’ section are mostly single-centered point patterns where the points show one central cluster of high density values. We also designed two comparative experiments to investigate how the number of centers affects the scale effect. As shown in Fig 9, we extended the single-centered simulated data (Fig 9(a)) to dual-centered patterns (Fig 9(b)) and calculated their *S*_*o*_. The *S*_*o*_ for dual-centered patterns when *N* = 10, 000 and 20,000 are both coarser than the *S*_*o*_ from single-centered patterns with the same *N*, which implies that the increase of the number of centers leads to a coarser *S*_*o*_. The *S*_*o*_ for the dual-centered pattern when *N* = 10,000 is coarser than that when *N* = 20,000. This is consistent with the conclusions from single-centered data regarding the impact of *N* on the optimized scale.

### Potential applications and limitations

The MAUP is a pervasive phenomenon for both intensive and extensive geographical data. However, there is insufficient research on how to quantify the scale effect caused by the MAUP. Due to the rapid development of ICTs, more and more individual-level crowd-sourced data are generated on a daily basis, and many of these datasets are point-based. To this end, this paper defined a series of indicators (e.g., the NSR and the *Hom* indicator) to quantify how the MAUP manifests itself when aggregating point data into areal units.

The methodology can be used to select the optimal scale for aggregation in various applications in geography. For example, similar to the Weibo case study, many researchers have used various types of point-based location data (e.g., social media check-in data, georeferenced mobile phone records, and taxi pick-up/drop-off data) to analyze the magnitude of human mobility in different urban districts [8, 16–18]. Research questions can range from basic summary statistics like “Which part of New York has the most Twitter check-ins during Christmas?” to a more complex one like “How should we quantify the mobility flows between urban regions based on taxi data?” In all these studies, a crucial data pre-processing step is to determine the size of the spatial unit for aggregating the point data. The proposed method provides a feasible way to quantitatively assess the influence of the scale of analysis units when applying crowd-sourced data to urban studies.

The application of the proposed method is not limited to using crowd-sourced mobility data in urban geography. In demographic studies, a remaining challenge is to mitigate the MAUP caused by aggregating population data based on different spatial units. In physical geography, a similar problem exists for animal tracking data, where researchers need to determine the optimal scale for aggregating location points from tracking devices [45]. This study takes a first step in providing a feasible solution to the aforementioned research problems.

The proposed method has several limitations. First, through trial and error, we found that this method does not work well when the data is very sparsely distributed. Sparsely distributed data shows no clear spatial pattern, so it is difficult to find a suitable model to fit the estimated semi-variance values. This may limit the application of this method to sparse datasets, such as taxi pick-up data after midnight or check-in data in a remote rural area. In addition, the quality of the check-in data may be influenced by various factors such as the representativeness of social media data and the strategy of data collection, which inevitably affect the results in the case study. Social media sites like Weibo are more likely to attract users with a certain demographic profile (e.g., young people), which leads to a biased sampling of the population. For the Weibo POI data used in this study, users are allowed to check in to a POI when they are within a certain distance of the location, which naturally leads to data accuracy issues. Because this study aims to propose a methodology instead of generating empirical results, we did not directly address the data quality issues. In practice, researchers should be aware of the influence of data quality issues on the results when applying our method to their own data.

## Conclusion

The scale effect is an important issue in geography. With the development of ICTs, massive high-resolution geo-tagged data is available for investigating human mobility patterns and the socioeconomic environment. Spatial aggregation is necessary to investigate collective patterns from individual-level big geo-data, and this inevitably leads to the challenge of selecting an optimal scale in spatial analysis.

We proposed a method to quantitatively evaluate the scale effect of extensive data, which is a common type of big geo-data. Because semi-variograms can provide rich spatial information at different lag distances, we employed the nugget-sill ratio as a quantitative measure to characterize the structure of spatial data variance at multiple scales. Two sets of simulated experiments showed that both very fine and very coarse scales lead to high NSR values, and a low NSR tends to appear at a medium scale. This observation is consistent with the structures of spatial variances. In addition, we defined the scale where the first local minimum NSR occurs as the optimal scale (*S*_*o*_), the results show that as *σ* (i.e., the dispersion of spatial data) increases, the *S*_*o*_ value gets coarser. The conclusion is consistent with our perception that a finer analysis unit is more appropriate for data with a higher spatial heterogeneity, otherwise a coarser scale is more suitable. It demonstrates the rationality of our method in quantifying the scale effect. We also used Weibo check-in data from four Chinese cities (Beijing, Shanghai, Chengdu, and Wuhan) as a case study. The results suggest that the optimal scale *S*_*o*_ for these cities are 600 m, 600 m, 900 m, and 700 m, respectively.

Overall, a very fine scale indicates that the analysis units are too small and there are not enough points to be aggregated in each unit; however, a scale too coarse will lead to over-generalization of the data and a substantial loss of information. Therefore, it is important to find a balance point between the level of detail and the degree of aggregation, which is the main contribution of this study. We adopted a classic geostatistical method (i.e., the semi-variogram) and provided a new perspective to quantify intra-unit variation and inter-variation at different scales. The method in this study offers a useful data processing strategy to optimize spatial scales when aggregating big geo-data in urban studies.

Geospatial big data have many potential issues, such as data sparsity, data representativeness, and other data quality issues, which can lead to non-stationary results. In fact, the semi-variogram contains much more information than we explored in this study and may be useful for improving our results in the future to optimize the scale of spatial analysis. In addition, the proposed method focuses on quantifying intra-unit variation when exploring the scale effect, but in practice, different datasets and urban studies may need to adopt different indicators based on their specific needs. Future studies should focus on expanding this framework by exploring the choice of indicators for different datasets.

## Supporting information

S1 File Weibo check-ins. Valid records of the POI check-in data in Beijing, Shanghai, Chengdu and Wuhan from Sina Weibo in 2014.
(ZIP)
